# Hystero-salpingography: An obituary or a new beginning…?

**Published:** 2008-05

**Authors:** Nitin P Ghonge, Sanchita Dube

**Affiliations:** Department of Radiology, Diwan Chand Imaging Research Center, New Delhi - 110 001, India; 1 Department of Obstetrics and Gynecology, Apollo Hospital, Sarita Vihar, New Delhi - 110 076, India. E-mail: drnitinghonge@rediffmail.com

Dear Sir,

With the advent of diagnostic and operative laparoscopy, the role and relevance of hysterosalpingography (HSG) as a routine investigation in infertility is gradually being debated. A recently published article based on a multicenter randomized controlled trial failed to show any statistically significant difference in cumulative pregnancy rates with the routine use of HSG in the workup of infertility.[1] According to us, there are a few technical lapses in the procedure and its interpretation, which result in its relatively poor performance as compared to laparoscopy, hysteroscopy, and salpingoscopy. This issue is now being globally understood and although this procedure has been around for about 100 years, “how-to-do” workshops on HSG were included in the annual conference of the Radiological Society of North America (RSNA) in 2004 and 2005. HSG, if performed and interpreted carefully, can offer a wealth of information.[2]

In view of this, the authors undertook a questionnaire-based study to analyze the prevalent clinical practices with respect to HSG, in Delhi and the National Capital Region (NCR) with the aim of predicting the cause of its relatively “poor” performance (*See Addendum*). The respondents included 50 radiologists and 50 gynecologists, irrespective of their practice settings and years of experience.

The predominant number of respondents preferred to use a metal cannula, which is known to cause more pain than a plastic cannula, during the stages of instrumentation, contrast injection and immediately post-procedure.[3] Pre-procedural antibiotics should always be advised.[4] Only 44% of respondents in this study, however, complied with this. As high as 40% of the respondents failed to realize that a slow gradual mode of contrast injection was the best technique to adopt, as this delineates the endometrial cavity in the non-distended state. This provides a unique opportunity to diagnose small subtle intra-endometrial space-occupying lesions or synechiae, which tend to get obscured in the late-distended phase. The patient should always be asked to empty her bladder before the procedure, to avoid discomfort during cannulation. The role of gentle psychological reassurance cannot be neglected.[5] The procedure should be explained to the patient in detail and no attempt should be made to force the catheter against resistance. The use of the Valsalva maneuver may be helpful during a difficult cannulation. After the images are acquired, a “pull-release” maneuver should always be performed. The relative change in position of the non-median uterus and the fallopian tubes gives vital clues about peritubal adhesions.[4] Interestingly, 78% respondents were not aware of these maneuvers.

Less than half of the respondents realized that the external uterine contour could also at times be evaluated on HSG, when the extravasated contrast delineates the uterine surface or when there is significant intravasation into the myometrial veins. This information about the external uterine contour helps diagnose underlying uterine anomalies.[6] A little more than one-third of the respondents realized that HSG was useful in predicting the reproductive outcome in patients with an arcuate uterus. The arcuate uterus ratio is the ratio of the depth of indentation and the intercornual distance. If the arcuate uterus ratio is less than 10%, an adverse reproductive outcome is not anticipated.[7]

The respondents were particularly concerned about tubal patency rather than mucosal details or peritubal disease, when it came to tubal imaging. Though, the term “tubal patency” is over-emphasized in HSG literature, the ideal terminology should be “tubal normalcy”. It should be ascertained that contrast spill is truly a “free” intraperitoneal spillage and not a “localized” spill. Peritubal venous intravasation is an important sign on HSG that may favor the likelihood of tubal disease. It would be worthwhile to reiterate here that, “free” intraperitoneal spillage is not the only parameter to establish tubal normalcy on HSG. Mucosal evaluation and the likelihood of peritubal adhesions are integral components of tubal assessment.[8]

The majority of respondents felt that the therapeutic effect of radiological contrast media was a myth rather than reality.[9] The awareness of selective salpingography (SS) and fallopian tube catheterization (FTC) procedures was also not widespread. About 72% of respondents were not aware that gametes are relatively radio-resistant as compared to zygotes. Radiation-related issues are certainly valid but these should not be over-emphasized and they do not constitute an ethical evidence to eliminate HSG from routine infertility work-up. The ideal practice would be to adopt an accurate technical protocol, which restricts the radiation dose to “as low as reasonably achievable” (ALARA). HSG should never be performed without a fluoroscopy unit. At least 39% of respondents did not realize that digital fluoroscopy decreases mean skin and ovarian dose as compared to analog systems.[10] Apart from these, the ability to save a particular fluoroscopy frame in a digital system with the automatic elimination of “spot film” acquisition helps in further dose reduction.

As revealed in this questionnaire-based analysis, there are definite “technical” and “interpretational” lapses in the clinical practice of the HSG. There are certain individual perceptions about HSG and a professional bias towards alternate options, which tend to drive the clinicians away from HSG. Radiation-related misconceptions are particularly significant. These technical lapses can certainly contribute to the “poor” performance of HSG, apart from its inherent limitations. A proper attention to technique will help make HSG a vital component in routine infertility investigations.

## Addendum

The study questionnaire used in the study is shown below, which included 35 objective questions apart from questions pertaining to the respondent's characteristics. The questions were based on procedural technique (13), image analysis (7), subject knowledge (6) and the perceptions/biases associated with HSG (9). The figures in bracket denote the number of questions in each category.

### 'How-you-do-it?' Questionnaire'

**Respondent characteristics**

1. Serial number:…………

2. Present designation: **Junior Resident/Senior Resident/Junior Consultant/Senior Consultant.**

3. Practice settings: **Institutional/Group/Individual. If institutional, Government/Private.**

4. Number of years in practice: <5 years/5-10 years/10-15 years/>15 years.

5. Average number of HSG procedures performed/interpreted in a month: <10/10-30/30-50/>50.

**Procedural Techniques: “What you routinely do?”**

6. Radiography equipment: **Over-couch (tube above the table) Radiography Machine/Under-couch (tube below the table) Image Intensifier/Digital Fluoroscopy System/Others (please specify)**

7. Who exactly explains the procedure to the patient: The one who **does cervical cannulation/does radiographic exposure/report the HSG.**

8. Type of analgesia: **Oral NSAID’s only/Oral NSAID's with Smooth Muscle Relaxants (SMR)/Oral NSAID's with SMR.**

9. Antibiotic prophylaxis: **Pre-procedural also/Post-procedural only.**

10. Instruments for cervical cannulation: **Metal Cannula/Plastic Cannula.**

11. Ensure that the instruments are out of the patient's view: **Always/Depends/Not really bothered.**

12. Use of Valsalva maneuver for difficult cannulation (for eq. posteriorly placed cervix): **Always/At times/ Never.**

13. Mode of contrast injection: **Slow gradual/Rapid abrupt/Bi-phasic.**

14. The radiographic exposure is made by a **trained Radiologist/trained Radiographer/Others.**

15. The first HSG spot often shows **peritoneal spillage/tubes/distended endometrial cavity/non-distended endometrial cavity/varies from patient to patient.**

16. Image analysis is done during **procedure itself/afterwards.**

17. After the procedure, “Perform pulling and releasing maneuver”: **Always/At times/What's that?**

18. At the end, calculate the radiation dose to patient: **Always/At times/Can't calculate in our set-up.**

**Image Analysis: “What is your opinion?”**

19. HSG never provides information about the external uterine contour: **True/False.**

20. If there is unilateral peritubal venous intravasation of contrast with ipsilateral peritoneal spillage in later spots of HSG: **There is definite possibility of distal tubal disease/Forget it! patency is patency.**

21. HSG can predict the prognosis in arcuate uterus: **True/False.**

22. Tubal mucosal evaluation is feasible with HSG: **Always/At times/Never.**

23. Peritubal adhesive disease cannot be diagnosed on HSG: **True/False.**

24. LSCS scar can be confused with **polyps/adhesions/fi broids/foreign body.**

25. Linear longitudinal folds can be normally seen in the endometrial cavity: **True/False.**

**Subject Knowledge: “What you know about this?”**

26. In proximal tubal disease, the tubal wall is involved early in the course of the disease: **True/False.**

27. What can be done to diff erentiate between tubal cornual occlusion and cornual spasm, apart from wait and smooth muscle relaxants? **Selective Salphingography/Salphingoscopy/Saline Infusion USG.**

28. What should be the location of the distal-most end of Cannula: **external os/internal os/mid-cavity/just proximal to fundus.**

29. In patients with proximal tubal occlusion, what are the treatment options other than Laparoscopy and Salphingoscopy? **FT catheterization/Tuboplasty.**

30. The radiation dose to skin and the ovaries are same with analog and digital fluoroscopy: **True/False.**

31. Tubal perfusion pressure is a potential predictor of the recanalization success: **True/False.**

**Biases and Perceptions: “What do you think?”**

32. The radiation dose to patient is **significantly/insignificantly** affected by the type of machine and the prevalent practice at the center.

33. Which of this two terms is more appropriate in context with HSG: **Tubal patency/Tubal normalcy.**

34. The gametes are relatively radio-resistant, as compared to the fertilized zygote: **True/False.**

35. A patent tube on HSG with peritubal adhesions will never interfere with fertility: **True/False.**

36. The force applied during contrast injection should be **gentle/proportional to the resistance.**

37. Immediately aft er an essentially normal HSG, it is always wise to go for Laparoscopy: **True/False.**

38. The therapeutic effect of the radiological contrast media in HSG is a **myth/reality.**

39. Unilateral patent tube (with peritubal adhesion) and contralateral proximal tubal occlusion: **Unilateral patency is sufficient, wait and watch/Not enough, treat the contralateral tube.**

40. In your opinion: **Is HSG Out-dated**, as a routine infertility investigation in the present era? **Yes/No.** (Give reasons)
